# Phytoremedial effect of *Withania somnifera* against arsenic-induced testicular toxicity in Charles Foster rats 

**Published:** 2015

**Authors:** Arun Kumar, Ranjit Kumar, Mohammad Samuir Rahman, Mohammad Asif Iqubal, Gautam Anand, Pintoo Kumar Niraj, Mohammad Ali

**Affiliations:** 1 *Mahavir Cancer Institute & Research Centre, Patna, Bihar, India*

**Keywords:** *Sodium arsenite*, *W. somnifera*, *Rats*, *Testicular toxicity*, *Sperm Count*, *Testosterone*

## Abstract

**Objective:**

The main objective of the current study was to observe the ameliorative effect of *Withania somnifera* on arsenic-induced testicular toxicity by exploring the crucial parameters such as sperm counts, sperm motility, hormonal assay and lipid peroxidation including histopathology.

**Materials and Methods:**

In the present study, arsenic in the form of sodium arsenite was administered orally to male Charles Foster rats for 45 days. Thereafter, ethanolic root extract of *Withania somnifera* was administered for 30 days to observe its ameliorative effect on male reproductive system.

**Results:**

The study revealed that after administration of sodium arsenite, there was a decrease in the sperm counts and sperm motility accompanied by an increased incidence of sperm abnormalities and hormonal imbalance leading to infertility. However, after administration of *Withania somnifera*, there was significant reversal in the parameters denoting that it not only possesses antioxidant and rejuvenating property but also maintains the cellular integrity of testicular cells leading to normal functioning of it.

**Conclusion:**

The study concludes that *Withania somnifera* possesses phytoremedial effect. It is one of the best antidotes against arsenic-induced reproductive toxicity.

## Introduction

Arsenic, the king of poisons, has probably influenced human history more than any other element or toxic compound. It is present in the environment and humans all over the world are exposed to small amounts, mostly through water, food, and air. However, the presence of high levels of arsenic in ground water, the main source of drinking water in many countries around the world, has drawn the attention of the scientific community. Millions of people are exposed to high levels of inorganic arsenic through drinking water. It has thus become a major public health problem in many countries in South and East Asia and a great burden on water supply authorities. The mechanism of arsenic accumulation in the Bengal Delta Plain is thought to have occurred during the late Quaternary age (Holocene age) with arsenic-containing alluvial sediments deposited by the Ganges, Brahmaputra. Meghna, and other smaller rivers that flow across the Bengal Delta Plain into the Bay of Bengal (Mukherjee and Bhattacharya, 2001 ▶). In the Bengal Delta Plain, the arsenic is adsorbed as arsenic oxyanions onto oxyhydroxides of iron, aluminium, and manganese and then mobilized in the alluvial aquifers where, due to the reducing environment, the oxyhydroxides are dissolved by biogeochemical processes, releasing the arsenic into the groundwater (Mukherjee et al., 2006 ▶)

Arsenite binds to cellular sulfhydryl and disrupts sulfhydryl containing enzymes (^Aposhian and Aposhian, 1989a ▶^; ^Aposhian, 1989b ▶^; Aposhian and Aposhian, 2006 ▶). It also inhibits numerous other cellular enzymes such as those which are essential for cellular glucose uptake, gluconeogenesis, and fatty acid oxidation and product – ion of glutathione through sulfhydryl group binding (Szinicz and Forth, 1988 ▶; Singh and Rana, 2007 ▶). Arsenite does not compete with phosphate, but tends to bind to dithiol groups, replacing the stable phosphorus anion in phosphate with the less stable As (V) anion lead to rapid hydrolysis of high energy bonds in compounds such as ATP, a process that leads to loss of high energy phosphate bonds and effectively “uncouples" mitochondrial respiration (Rossman, 2007 ▶).

The second major toxicity caused by arsenic is termed as “arsenolysis” in which oxidative phosphorylation is hampered as arsenic binds with ADP to form ADP arsenate which irreversibly results in loss of energy in cells. Thus, ROS’s are capable of damaging DNA, lipids, and proteins and finally, hampering the cell signalling (Yang and Frenkel, 2002 ▶; Qian et al., 2003 ▶; Singh et al., 2011 ▶).

Developmental effects, cancer, and cardiovascular diseases have all been associated with long-term exposure to arsenic in humans. There is a paucity of epidemiological studies of arsenic reproductive toxicity, and there are only a few of arsenic developmental toxicity (DeSesso, 1998 ▶; Tchounwou et al., 2003 ▶). Epidemiological studies have reported that arsenic exposure in uterus increased spontaneous abortion and stillbirth and decreased birth weight (Ahmad et al., 2001 ▶; Ihrig et al., 1998 ▶; Milton et al., 2005 ▶). However, these studies lack detailed information on confounders (exposure to other metals, smoking, maternal age, etc.) and accurate maternal arsenic exposure. The use of animal models therefore is essential for investigating arsenic developmental and reproductive toxicities, because it allows greater experimental controls, including the confounders mentioned above, and direct observations of developmental changes before birth. Furthermore, it provides opportunities to investigate the effects of interventions that have not been approved for humans. 

*Withania somnifera* also called asAshwagandha, winter cherry, or Indian Ginseng has been an important medicinal herb in Ayurveda and indigenous medical systems for over 3000 years. Studies indicate that Ashwagandha possesses antioxidant (Dhuley, 1998 ▶), anti-inflammatory (Begum and Sadique, 2004 ▶), immunomodulatory (Rasool and Varalakshmi, 2006 ▶), antitumor (Jayaprakasam et al., 2003 ▶), antistress (Vaishali et al., 1987 ▶), adaptogenic (Bhattacharya and Muruganandam, 2003 ▶), antiulcer (Bhatnagar et al., 2005 ▶), and rejuvenating properties (Kumar et al., 2013 ▶ and 2015, and; Patil et al., 2012 ▶). However, no studies has reported the effect of *W.somnifera *root extract as antidote against arsenic-induced male reproductive toxicity in rats. 

Thus, present study deciphers the ameliorative effect of *W.somnifera *against arsenic induced testicular toxicity.

## Materials and Methods

**Test chemical**

Sodium arsenite AR (98.0%), (CAS No.7784-46-5, Lot No. 20/21-28a-45-60-61) manufactured by Loba Chemie, India was obtained from the scientific store of Patna. 

**Animals**

Charles Foster rats (30 males), weighing around 160-180g of approx 8 weeks old, were obtained from animal house of Mahavir Cancer Institute and Research Centre, Patna, India (CPCSEA Regd-No. 1129/bc/07/CPCSEA). The research work was approved by the IAEC (Institutional Animal Ethics Committee) with IAEC No. IAEC/2012/12/04. Food and water to rats were provided *ad libitum* (prepared mixed formulated food by the laboratory itself). The experimental animals were housed in conventional polypropylene cages in small groups (2 each). The rats were randomly assigned to control and treatment groups. The temperature in the experimental animal room was maintained at 22±2^o^C with 12 h light/dark cycle.

**Preparation of plant ethanolic extract**

In the present study, dry root of *W.somnifera* was purchased from Haridwar Medicinal Store, Haridwar, Uttrakahand, India. The identity of the medicinal plant was confirmed by Dr. Ramakant Pandey (Botanist), Department of Biochemistry, Patna University, Patna, Bihar, India. The collected root of *W.somnifera *was shade dried and grinded into fine powder. The powder was then soaked in 70% ethanol for 48 hours and finally extracted with absolute ethanol using Soxhlet apparatus running for 6-8 hours and the residue was then concentrated and dried at 37^o^C. The ethanolic extract dose was calculated after LD_50_ estimation and finally made to 100 mg kg^-1^ body weight.

**Experimental design**

In the present study, arsenic in the form of sodium arsenite was administered orally to male Charles Foster rats (n = 24) at the dose of 8 mg Kg^-1^ body weight per day up to 45 days while control male rats (n = 6) were also taken for the comparative study. Thereafter, ethanolic root extract of *Withania somnifera* (Ashwagandha) was prepared and administered at the dose of 100 mg Kg^-1^ body weight per day for up to 30days upon 45 days sodium arsenite pre-treated group to observe its ameliorative effect. Drinking water and feed was provided to the animals *ad libitum*. After completion of the antidote treatment, rats were anaesthesized, their sperm were counted and then they were sacrificed and their serum was extracted for lipid peroxidation study, luteinising hormone and testosterone hormone assay while their testes were fixed in the neutral formalin for histopathological study.

**Sperm counts**

The cauda epididymis was dissected out and washed thoroughly in normal saline (0.85%). Then, it was incised and made puncture at several places in 1 ml of distilled water in watch glass so as to allow the sperm to ooze out. After that, two drops of eosin Y was mixed well with the sperms. Sperm count was done using an improved Neubauer’s chamber. A drop of the above preparation was taken onto it and observed at 450x magnification. 

**Sperm motility**

Cauda epididymis was dissected out and ruptured on microscopic slide. After covering it with a cover slip, the motility of the spermatozoa was examined. 

**Hormonal assay**

Using the ELISA method, luteinising hormone (LH) and testosterone were measured by a kit purchased from LILAC Medicare (P) Ltd., Mumbai, India. The normal range was calibrated and then 25μl serum samples were taken in the microwell plates. One hundred μl of enzyme conjugate was added in each well and left for incubation at 37^o^C in incubator for 1 hour. Then, the wells were washed with 300μl distilled water for at least three times and blotted. Then, 100μl TMB solution was added as substrate into each well plate and was again left for the incubation for 15 minutes for the color development. Finally, 100μl stop solution was added into each well to stop the reaction. Reading was taken out at 630nm through Merck ELISA reader in ng/ml value. 

**Lipid peroxidation**

Thiobarbituric acid reactive substances (TBARS), as a marker for LPO, were determined by the double heating method (Draper & Hadley, 1990 ▶). This method is a spectrophotometric measurement of the color produced during the reaction to thiobarbituric acid (TBA) with malondialdehyde (MDA). For this purpose, 2.5 ml of 100 g/l trichloroacetic acid (TCA) solution was added to 0.5 ml serum in a centrifuge tube and incubated for 15 min at 90^o^C. After cooling in tap water, the mixture was centrifuged at 3000 g for 10 min, and 2 ml of the supernatant was added to 1 ml of 6.7 g/l TBA solution in a test-tube and again incubated for 15 min at 90^o^C. The solution was then cooled in tap water and its absorbance was measured using Thermo Scientific UV-10 (UV–Vis) spectrophotometer (USA) at 532 nm. 

**Histopathology**

All of the rats were sacrificed after the scheduled period. A midsaggital incision was made and testicular tissue from all of the rats were removed and fixed in 10% neutral formalin. For the light microscopic study, the haemotoxylin and eosin stained slides were prepared and the sections were viewed under light microscope. 

**Statistical analysis**

Results are presented as mean ± SD and total variation present in a set of data was analysed through one way analysis of variance (ANOVA). Difference among mean values has been analyzed by applying Dunnett’s test. Calculations were performed with the Graph Pad Prism Program (Graph Pad software, Inc., San Diego, U.S.A.). The criterion for statistical significance was set at p< 0.05.

## Results

**Morbidity and mortality**

The rats after arsenic exposure (8mg Kg^-1^ body weight) for 45 days showed signs of toxicity such as nausea, nose bleeding, lack of body co-ordination (11 percent of rats showed paralysis like symptoms), blackening of tongue and foot, and general body weakness.

**Sperm counts**

**Figure 1:**
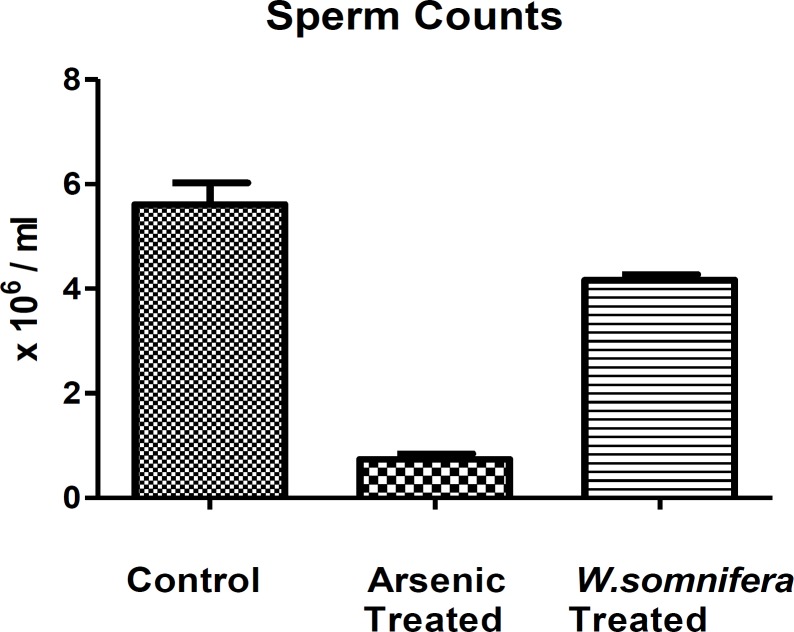
Showing Sperm counts in different groups in which *W.somnifera *shows significant amelioration. The data are presented as mean ± S.D, n = 6, significance at p< 0.001

The sodium arsenite-treated rats caused marked reduction in their sperm counts in comparison to control ones. However, after the administration of *W.somnifera, *there was a significant increase in the sperm counts denoting normalisation in the testicular functioning (Figure.1.). 

**Sperm morphology and motility**

**Figure 2:**
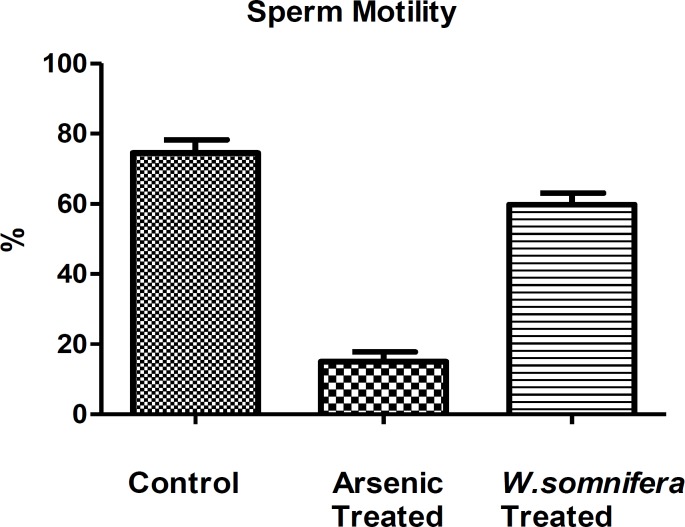
Showing Sperm motility in different groups in which *W.somnifera *shows significant amelioration. The data are presented as mean ± S.D, n = 6, significance at p< 0.001

The sodium arsenite-treated rats caused marked reduction in sperm motility in comparison to control ones (Figure 2). The major sperm abnormalities observed were loss of sperm tails, coiling in sperm tails, etc. denoting the degeneration caused by sodium arsenite. However, after the administration of *W.somnifera *there was a significant increase in the sperm motility denoting restoration in the spermatozoa.

**Hormonal assay**

**Figure 3:**
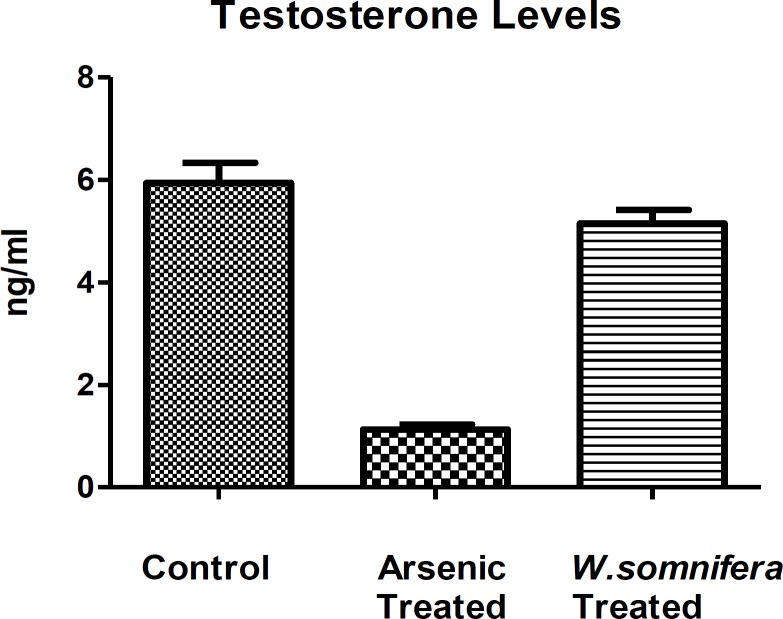
Showing Testosterone hormone levels in different groups in which *W.somnifera *shows significant amelioration. The data are presented as mean ± S.D, n = 6, significance at p< 0.001

**Figure 4:**
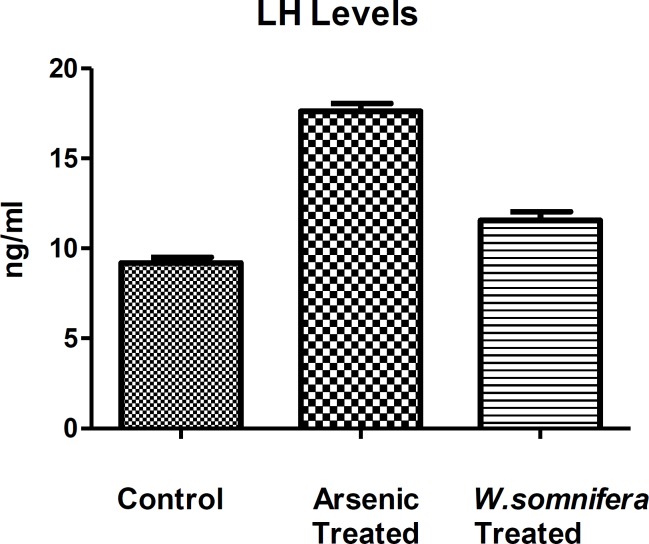
Showing Luteinising hormone levels indifferent groups in which *W.somnifera *shows significant amelioration. The data are presented as mean ± S.D, n = 6, significance at p< 0.001.

The serum testosterone level showed decreased levels after arsenic exposure in comparison to control denoting the endocrine disruption while there was a significant increase in its level after administration of *W.somnifera *(p<0.001) denoting the normalization in the endocrine function (Figure 3). Whereas, there was an increase in the LH levels after arsenic exposure in comparison to control but there was as decrease in the LH levels (p<0.001) after administration of *W.somnifera *(Figure 4).

**Lipid peroxidation assay**

**Figure 5:**
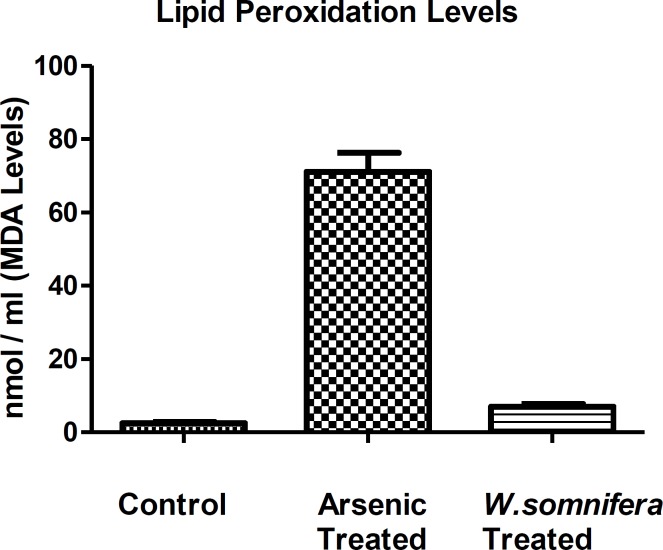
Showing Lipid peroxidation levels in different groups in which *W.somnifera *shows significant amelioration. The data are presented as mean ± S.D, n = 6, significance at p< 0.001

There is an increase in the lipid peroxidation levels after arsenic exposure in comparison to control denoting the cellular oxidative stress but after the administration of *W.somnifera*, there was a significant decrease in the LPO levels denoting the antioxidant property of *W. somnifera *(Figure.5.). 

**Histopathological study**

**Figure 6:**
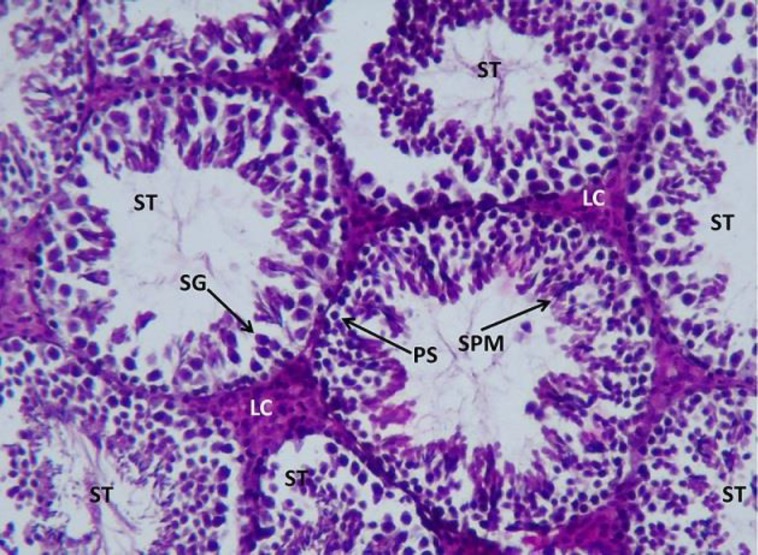
Light microphotograph (Haemotoxylin – Eosin (H&E) stained) section of control testis showing normal architecture of seminiferous tubules (ST) with well-arranged spermatogenetic stages – primary spermatocytes (PS), spermatogonia (SG), spermatids (SPM) and spermatozoa x 800

The control testis showed normal architecture of seminiferous tubules (ST) with well-arranged spermatogenetic stages –primary spermatocytes (PS), spermatogonia (SG), spermatids (SPM), and spermatozoa. The leydig cells aligning the inter–seminiferous tubules were normal denoting the normal functioning of the spermatogenesis (Figure.6.). 

**Figure 7:**
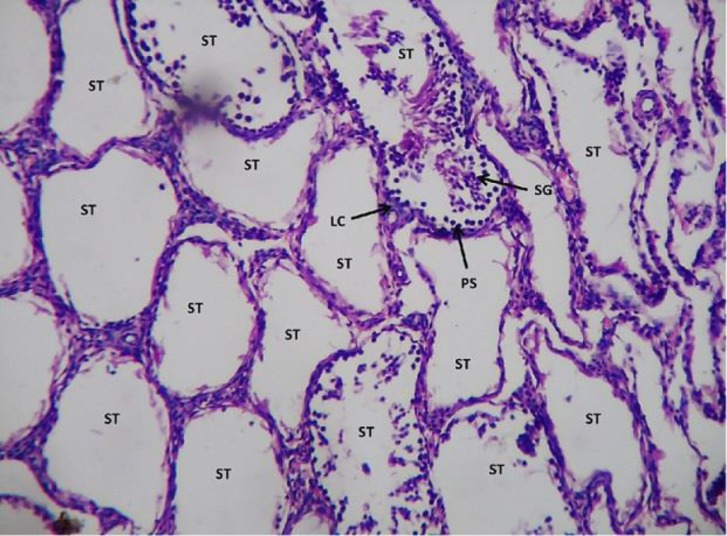
Light microphotograph (H&E stained) section of sodium arsenite treated testicular section showing severe degeneration. Seminiferous tubules with no spermatogenetic stages or if are present, only 5% denotes the normal functioning. The leydig cells are in highly degenerative condition as hemorrhage in them is observed x400.

**Figure 8:**
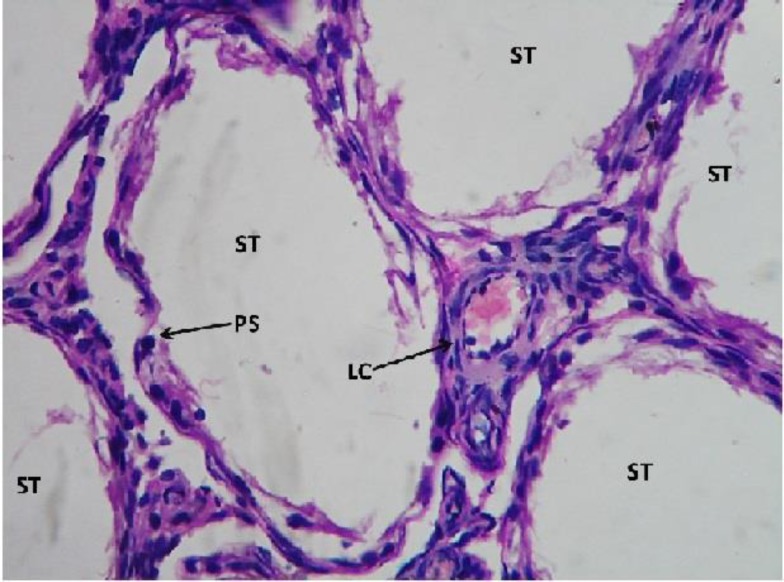
Showing magnified view of fig.7 x 800

However, in sodium arsenite-treated testicular sections, it caused severe damage to the testicular cells. The seminiferous tubules showed only 5% of the functioning of the testicular cells. The leydig cells, also appeared to be in highly degenerative condition as hemorrhage was observed within them (Figures.7 and 8).

However, after the administration of *W. somnifera *there was immense amelioration, as restoration in the spermatogenetic stages could be observed. 

**Figure 9:**
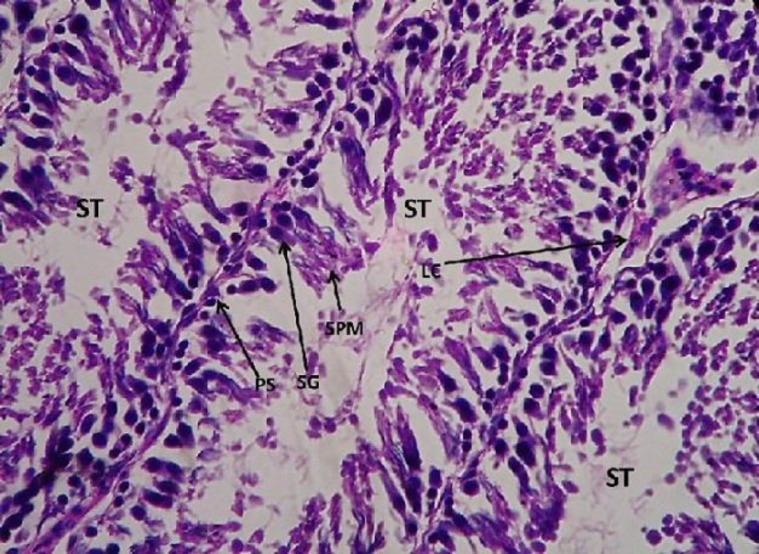
Light microphotograph (H&E stained) section of testicular cells with *W.somnifera * administered upon sodium arsenite pretreated showing immense amelioration as the primary spermatocytes (PS), spermatogonia (SG), spermatids (SPM) and spermatozoa all are well arranged denotes the significant normalisation in the function of the testicular cells. The leydig cell also shows amelioration x 800

**Figure 10:**
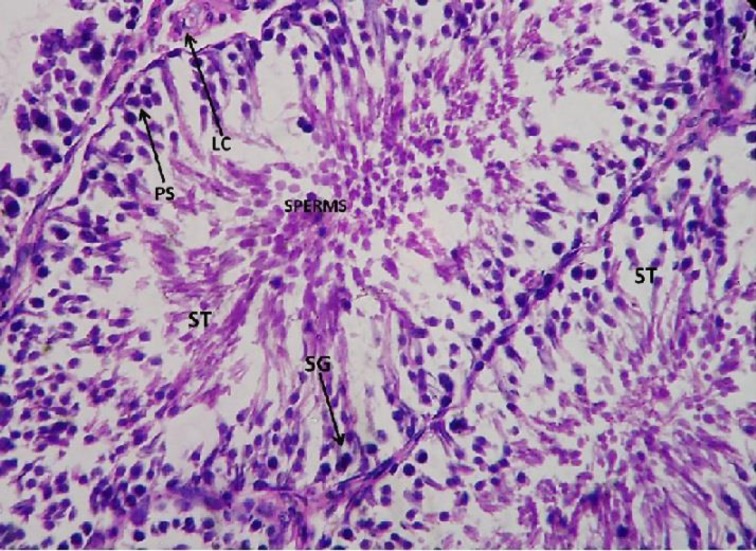
Light microphotograph (H&E stained) section of testicular cells with *W.somnifera * administered upon sodium arsenite pretreated showing immense amelioration as the primary spermatocytes (PS), spermatogonia (SG), spermatids (SPM) and spermatozoa all are well arranged denotes the significant normalisation in the function of the testicular cells. The leydig cell also shows amelioration x900

The primary spermatocytes (PS), spermatogonia (SG), spermatids (SPM), and spermatozoa all were well-arranged denoting the significant normalization in the function of the testicular cells. The leydig cells also showed amelioration denoting the normalization in its function (Figures.9 and 10).

## Discussion

In the present investigation, the arsenic exposure (8mg Kg^-1^ body weight) caused severe damage to the male reproductive function. The As III in sodium arsenite had deleterious effect on the vital tissues. The decreased levels of sperm counts, sperm motility, testosterone levels, and lipid peroxidation denotes that arsenic caused release of free radicals which in turn caused endocrine disruption which led to the high secretion of luteinizing hormone (LH) leading to improper functioning of the leydig cells. The extreme low level of testosterone has caused abnormal functioning of the spermatogenesis as no sperm was observed in the lumen of seminiferous tubules in the histopathological findings. Altogether, this study reveals that, arsenic caused arrest of spermatogenesis in testicular cells leading it to male infertility. 

Similarly, other studies have found that arsenite exposure causes male reproductive toxicity when administered through drinking water or intraperitoneally (Chinoy et al., 2004 ▶). AsIII interferes with spermatogenesis and alters the activities of spermatogenic enzymes (Pant et al., 2004 ▶). Furthermore, As III lowers the levels of testosterone and gonadotropin. Arsenic may act on the brain or pituitary gland as well as directly on the germ cells (Sarkar et al., 2003 ▶). Male mice exposed to sodium arsenite in drinking water showed reproductive toxicity without clinical effects (Pant et al., 2001 ▶). 

In mice, in addition to spermatogenesis, cholesterol metabolism and testicular testosterone level were also affected by AsIII (Chinoy et al., 2004 ▶). Male Swiss mice when treated with arsenic trioxide (As_2_O_3_) showed increased cholesterol levels and decreased protein levels in the testes. Testicular structural damage observed included degeneration of tubules and denudation of germinal epithelial cells. There was also lack of sperm in the lumen of seminiferous tubules. In addition, testicular activities of 3*β*- HSD, 17*β*-HSD, and testosterone levels in the serum were decreased. In the testis, cholesterol in the interstitial tissue is used for testosterone synthesis (Kabbaj et al., 2003 ▶). 

 Another study emphasized that when rats were treated with sodium arsenite, there happened a significant decrease in their relative testicular weight, accessory sex organ weights, and epididymal sperm counts. The same was true for plasma concentrations of luteinizing hormone (LH), FSH, and testosterone. Because, late stages of spermatogenesis were especially sensitive to testosterone, quantitative analysis of spermatogenesis was carried out by counting the relative number of each variety of germ cells at stage VII of the seminiferous epithelium cycle (Leblond and Clermont, 1952 ▶). Massive degeneration of all germ cells at stage VII was observed. These authors also suggested the observed arsenic-induced low levels of LH and FSH might be the trigger of suppressed testosterone synthesis. Low testosterone consequently increased spermatid degeneration. Although AsIII may act on the brain or pituitary to suppress LH and FSH levels, direct inhibition on germ cells by binding to thiol cannot be ruled out. Another possible cause of the reduction in serum LH, FSH, and testosterone levels could be the high serum corticosterone levels. High corticosterone can reduce serum gonadotropin and testosterone levels (Hardy et al., 2005 ▶; Vreeburg et al., 1988 ▶) and has been reported in AsIII-treated rats (Wang et al., 1994 ▶). However, when the same was assayed in rat blood serum, increased LH levels were shown (Ali et al., 2013 ▶; Reddy et al., 2010 ▶). 

The most fascinating finding of the present study is the amelioration in the male reproductive function by *W. somnifera *as an antioxidant, with its active constituents sitoindosides VII-X and withaferin decreased the levels of lipid peroxidation (Ahmad et al., 2010 ▶; Bhatnagar et al., 2005 ▶; Gupta et al., 2003 ▶; Bhattacharya et al., 2001 ▶; Bhattacharya et al., 1997 ▶). *W.somnifera *is thought to be amphoteric and can help to regulate important physiological processes. When there is an excess of a certain hormone, the plant-based hormone precursor occupies cell membrane receptor sites in such a way that the actual hormone cannot attach and exert its effect normalising the hormonal activity (Ilayperuma et al., 2002 ▶; Abdel-Magied et al., 2001 ▶). Thus, the LH and testosterone levels were normalised. 

As traditionally, in Ayurveda as Rasayana, *W.somnifera *is used as rejuvenative tonic (Sukh Dev, 2005 ▶; API, 2001 ▶; Upton, 2000 ▶) and male reproductive booster (Shukla et al., 2011 ▶). In the present study, sodium arsenite caused low sperm counts, sperm motility, degeneration in sperm morphology, and complete arrest of spermatogenetic stages leading to infertility. However, after the administration of *W.somnifera*, there was a huge reversal in the testicular function as observed by normalization in the spermatogenetic stages. This denotes the rejuvenating activity of *W.somnifera* in testicular cells. Thus, from the entire study, it can be concluded that *W.somnifera *is the novel drug which not only possesses antioxidant and rejuvenating property but also maintains the cellular integrity of testicular cells leading to normal functioning of it. It is one of the drugs which can be used as an antidote against arsenic-induced reproductive toxicity.
